# Changes in the Composition of Unstimulated and Stimulated Saliva Due to Chewing Sour Cherry Gum and a Toothbrush Change

**DOI:** 10.3390/cells13030251

**Published:** 2024-01-29

**Authors:** Boglárka Emese Skopkó, Judit Rita Homoki, Mónika Éva Fazekas, Melinda Paholcsek, Péter Fauszt, Péter Dávid, László Stündl, Piroska Bíróné Molnár, Ildikó Noémi Forgács, Judit Váradi, Kinga Ágnes Bágyi, Judit Remenyik

**Affiliations:** 1 Department of Dentoalveolar Surgery, Faculty of Dentistry, University of Debrecen, 4032 Debrecen, Hungary; skopko.boglarka@dental.unideb.hu; 2 Institute of Food Technology, Faculty of Agricultural and Food Sciences and Environmental Management, University of Debrecen, 4032 Debrecen, Hungaryfazekas.monika@agr.unideb.hu (M.É.F.); paholcsek.melinda@med.unideb.hu (M.P.); david.peter@agr.unideb.hu (P.D.); stundl@agr.unideb.hu (L.S.); molnar.piroska@agr.unideb.hu (P.B.M.); forgacs.ildiko@agr.unideb.hu (I.N.F.); 3 Department of Pharmaceutical Technology, Faculty of Pharmacy, University of Debrecen, 4032 Debrecen, Hungary; varadi.judit@pharm.unideb.hu; 4 Department of Operative Dentistry and Endodontics, Faculty of Dentistry, University of Debrecen, 4032 Debrecen, Hungary; bagyi.kinga@dental.unideb.hu

**Keywords:** tumor necrosis factor α, Interleukin-1β, Mucin5B, Mucin7, oral microbiome, anthocyanin, toothbrush change

## Abstract

Background: Our previous studies demonstrated that sour cherry anthocyanins (AC) reduce the salivary count of *Streptococcus mutans* and inhibit salivary amylase activity within 30 minutes after chewing AC gum. AC gum and changing toothbrushes after scaling reduced the Gram-negative species in the unstimulated salivary microbiota. The present study examined the effect of AC gums on salivary factors, including changes in microbiome. Methods: The study was conducted over three weeks with two groups; young adults (18–30) and adults (30–45). Ten participants changed their toothbrushes, while the other 10 participants did not change after the control period. After scaling, all participants received three doses of AC gum daily. The salivary mRNA and protein levels of cytokines, mucins, melatonin, and the microbiota of unstimulated and stimulated saliva were determined by polymerase chain reaction, enzyme-linked immunosorbent assay, and 16S rRNA gene sequencing. Results: Significantly higher levels of tumor necrosis factor α (TNFα), interleukin-1β (IL-1β), mucin5B (MUC5B), mucin7 (MUC7), and melatonin were detected in stimulated saliva. Correlation analysis of these factors with the microbiota showed positive correlations with the genera *Lachnospiraceae*, *Eikenella*, *Saccharibacteria*_(TM7), *Streptococcus*, *Prevotella*, and *Haemophilus*. Conclusions: AC chewing gum has a beneficial effect on the composition of the oral microbiome, and toothbrush replacement leads to changes in the levels of salivary pro-inflammatory cytokines.

## 1. Introduction

Proper oral hygiene practices are important for maintaining good oral health as well as overall systemic well-being. Over the past decades, studies on the human microbiome have underscored the critical role of the oral microbiota in both health and disease. Therefore, the oral microbiome plays a pivotal role not only in oral health but also in systemic health. For example, the oral microbiota is directly involved in the aetiopathology of dental caries [1]. Recently proposed integrated hypothesis emphasizes the restoration of balance and maintenance of the microbial ecosystem in the oral cavity during the prevention and treatment of caries [2]. In this regard, saliva is the primary medium that helps maintain the ‘sialo-microbial-dental complex’ equilibrium in oral cavity [3]. 

Saliva constitutes factors crucial for the protection of teeth, maintenance of commensal oral microbiota, and systemic health. Salivary mucins, for instance, play pivotal roles in facilitating the adhesion [4], and growth of oral commensal bacteria [5], thereby contributing to the maintenance of the sialo-microbial-dental equilibrium [6,7,8]. MUCIN 5 subtype B (MUC5B) can selectively attach microorganisms (e.g., *Haemophilus parainfluenzae, Streptococcus mutans (S. mutans)*, and *non-MS Streptococci*) and is capable of keeping them in a planktonic form. In addition it also facilitates the coexistence of different microorganisms such as *S. sanguinis* and *S. mutans* [5]. MUCIN 7 (MUC7) attaches most of the *Streptococci* (*S. sanguinis, S. sobrinus,* and *S. oralis*) through the sialic acid side-chains [9], but it connects to *S. gordonii* through a trisaccharide in its major oligosaccharide structure [10]. Mucins have prebiotic function that helps their proliferation of commensal microbiota [5,6,11,12,13]. The production of the two secreted mucins is regulated by different pathways [7]. The gene of MUC5B is located on the 11p15.5 chromosome, and its expression can be activated by some pro-inflammatory cytokines, interleukin-1β (IL-1β), interleukin-6 (IL-6), and tumor necrosis factor α (TNFα) or by bacterial lipopolysaccharide (LPS) [14]. The transcription of MUC7 is activated through the nuclear factor-κB (NF-κB) pathway and induced by the production of TNFα [9].

The production of pro-inflammatory cytokines is a complex process that is induced by the immune response to oral microorganisms (e.g., bacterial lipopolysaccharide (LPS)). The interactions between different cytokines, microorganisms, and mucins can be observed as the level of IL-1β and the number of *S. mutans* counts were found to correlate with each other [15]. The level of TNFα and local production of IL-1β can enhance the production of IL-6 in saliva [16]. Furthermore, a higher level of IL-6 and TNFα can be observed in patients with active caries lesions [17]. After the treatment of carious lesions, a significant decrease could be observed in the level of IL-6 [18]. There is limited evidence implicating IL-2 in dental caries [16]. Overall, salivary biomarkers have been proposed as predictors of dental caries [19]. 

Melatonin, produced in the pineal gland, is an endogenous antioxidant that is also excreted in the saliva. Previous studies have demonstrated a higher level of melatonin in subjects without caries compared to those with caries [20]. Salivary melatonin inhibits TNFα and has an anticariogenic effect through the inhibition of matrix metalloproteinases [21]. Salivary amylase plays an important role in the neutralization of dietary carbohydrates and acids produced by the cariogenic bacteria [22]. The calcium (Ca^2+^) ions are able to stabilize the structure of amylase [23], thereby help reducing demineralization. The salivary proteins also aid in the remineralization of dental enamel by the attachment of Ca^2+^ ions [1]. The stimulated saliva contains more Ca^2+^, which is more supersaturated with hydroxyapatite and shows a higher remineralization capacity [24]. 

In our previous study, we demonstrated that chewing gum containing sour cherry anthocyanins (ACs) and changing the toothbrush reduced *S. mutans* counts and α-amylase activity in saliva [25]. In the present study, we investigated the effects of daily chewing of AC gum three times over two weeks and toothbrush replacement in young adults (AG I, 18–30) and adults (AG II, 31–45). We assessed changes in salivary mucins, pro-inflammatory cytokines, melatonin in terms of gene expression and protein expression. Moreover, correlation analysis of these proteins was performed in relation to the salivary microbiome. 

## 2. Materials and Methods

### 2.1. Study Participants

Ethical permission was accepted by ETT TUKEB, Hungary (license number: IV/1120-1/2020/EKU) and recommended by the DE KK RKEB/IKEB (Regional Ethical Committee) (protocol number: 5379-2019) at the University of Debrecen based on the principles of the World Medical Association Declaration of Helsinki. A study registration on URL: https://clinicaltrials.gov/ (accessed on 6 March 2022) was also made under the number 2022-IV/1120-1/2020. This was an open-label, self-controlled, multi-center study conducted between the Faculty of Dentistry, the Center for Complex Systems and Microbiome Innovations (KRÉMK), and the Faculty of Pharmacy at the University of Debrecen.

The participants were between the ages of 18 and 45 with good oral hygiene and good general health. The cohort was divided into two age groups: the members of age group I were between 18 and 30 years old, and the members of age group II were between 30 and 45 years old. 

Exclusion criteria include smoking, antibiotic treatment or infective disease during the past two months, diagnosed hyposalivation, oral infection (with visible signs), serious systemic disease, mental problems, periodontal disease, pregnancy, taking oral anticoncipiens, allergy to lactose. Inclusion criteria were age older than 18 years, signed consent statement, and good oral hygiene [26]. All of the participants signed the consent statement after an explanation of the study conditions. The participants were instructed to promptly notify us of any antibiotic or non-steroidal anti-inflammatory drug (NSAID) treatments. The participants’ number and group composition were the same as reported in our earlier work [27]. 

### 2.2. Study Design

**Figure 1:**
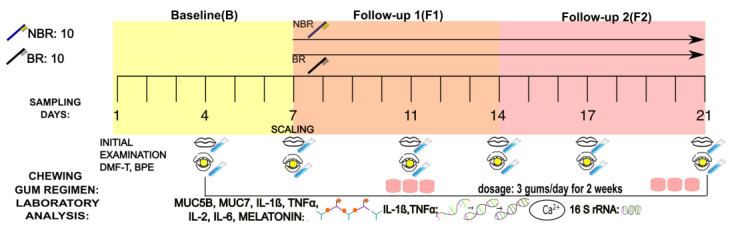
The sampling protocol of the 3-week study: control (baseline—B), 1st week of chewing the AC gum (follow-up 1—F1), and 2nd week of chewing the AC gum (follow-up 2—F2). NBR group, who did not change their toothbrush after calculus removal; BR group, who changed their toothbrush. After the initial tests (DMF-T status, BPE), during the first sampling occasion, the samples were taken on Days 1, 4, and 7 of each test week. The tube next to the closed mouth indicates unstimulated saliva, and the tube next to the chewing gum in the mouth indicates stimulated saliva sampling appointments. The chewing gum containing AC was continuously chewed 3 times a day for 2 weeks. Laboratory analysis: ELISA (enzyme-linked immunosorbent assay) is indicated by an antigen-antibody detection drawing for the determination of mucin-5B (MUC5B), mucin 7 (MUC7), interleukin-1β (IL-1β), tumor necrosis factor α (TNFα), interleukin-2 (IL-2), interleukin-6 (IL-6), and melatonin levels. PCR (polymerase chain reaction) is indicated by an RNA transcription drawing for the measurement of interleukin-1β (IL-1β) and tumor necrosis factor α (TNFα). Ca^2+^ levels (shown in a circle) were determined in the same way as in our earlier study. 16S gene sequencing (indicated by a DNA sequence) was performed only on Day 4 for B, Day 11 for F1, and Day 21 for F2.

The design of the study is depictured in Figure 1. Two patient groups (n = 10 each) were formed according to the age of the participants: young adults (18–30) and adults (30–45) (Figure 1).

The study was a self-control experiment, combined from baseline (B), first-week follow-up 1 (F1), and second-week follow-up 2 (F2), conducted by chewing gum with sour cherry extract.

During the selection of the participants, the DMF-T status of the patients was recorded on a pre-prepared status sheet. The BPE (Basic Periodontal Examination) index was used to rule out periodontal disease. The caries status was recorded based on the DMF-T (decayed, missing, and filled teeth).

The sampling occasions were on Day 1, Day 4, and Day 7 of the study weeks (where Day 7 of a week was the same as Day 1 of the forthcoming week during the 2-week chewing gum usage) between 11:00 a.m. and 14:00 p.m. based on the study protocol in our earlier experiment, which considered the circadian rhythm of the body when determining the sampling hours [27].

The first week of the study was the baseline (B) period. The second week was the F1 phase, when scaling occurred after the first sampling, followed by administration of the sour cherry chewing gum, and the third week was the F2 phase. Before the first administration of the gum, general well-being, DMF-T status, and a basic periodontal examination for each study participant were recorded on a status chart containing rubrics for the DMF-T status, basic periodontal examination, and plaque and calculus indices [28].

The participants received their chewing tablets at the beginning of each week. After the control (baseline, B) period, scaling occurred on Day 7 of the control week, followed by the participants receiving the sour cherry chewing gum. They were asked to chew the gum 3 times per day for 1–5 min after brushing their teeth, followed by eating the main dishes, for a period of 2 weeks. The stimulated saliva samples were taken at a different time following the same protocol as the unstimulated samples that were supplemented by chewing gum without an active (AC) ingredient during the 3-week experimental period. Half of the participants (n = 10) were asked to change their toothbrush after scaling, and the other half (n = 10) did not change their toothbrush after scaling.

### 2.3. Collection of Mixed Unstimulated and Stimulated Saliva 

The subjects were requested not to eat, drink, or use any chewing gum an hour before the sampling to avoid any stress factors influencing the production of unstimulated saliva [29,30]. The patients received all the information written on a fact sheet. A total of 1–1 mL of saliva was taken from each patient at each sampling time and sent to the Center for Complex Systems and Microbiome Innovations labs, where the samples were frozen at −80 °C for later processing. The saliva samples were transferred into RNAse-free tubes for sequencing in the same way as described in our previous study [27].

### 2.4. Preparation of the Chewing Gum with Sour Cherry Extract

*Chewing gum preparation:* Sour cherry chewing gum and placebo chewing gum were produced in the Department of Pharmaceutical Technology, which is licensed to produce chewing gum containing sour cherry extract. The sour cherry gum used in this investigation was prepared in the same way as described in our previous studies, Homoki et al. [25] and Skopko et al. [27]. Based on our earlier work, the main ingredients of the chewing gum were Geminis T BHA gum (Cafosa) base, xylitol, citric acid, glycerol, saccharine (Sigma), peppermint volatile oil, and sour cherry extract [27]. The flavoring ingredients (saccharine, citric acid, and glycerol) were dissolved in purified water. In the case of the sour cherry gum, 0.1 g of the anthocyanin-containing sour cherry extract was also added to this mixture. The emulsifying of the water phase was made into the melted gum stock at 60 °C, and then the peppermint oil was mixed in at 40 °C. The gum material was blocked out into 2.5 g pelleted tablets. Finally, after controlling the room temperature for 12 h, the pellets were placed into paper boxes and stored at 8–15 °C [25,27].

### 2.5. ELISA Methods

#### 2.5.1. Determination of MUC5B Concentration

MUC5B was measured with a sandwich immunoassay, the Enzyme-Linked Immunosorbent Assay Kit (MyBioSource, Inc., San Diego, CA, USA), following the instructions of the manufacturer (Number: MBS2024599 96) with SPECTROstar Nano (BMG Labtech GmbH, Ostenberg, Germany). The levels of MUC5B were reported in ng/mL of saliva. The detection ranges of MUC5B were 0.312–20 ng/mL.

#### 2.5.2. Determination of MUC7 Concentration

MUC7 was determined with a sandwich immunoassay, the Enzyme-Linked Immunosorbent Assay Kit (MyBioSource, Inc., San Diego, CA, USA), following the instructions of the manufacturer (Number: MBS762777) with SPECTROstar Nano (BMG Labtech GmbH, Ostenberg, Germany). The levels of MUC7 were reported in ng/mL of saliva. The detection ranges of MUC7 were 0.313–20 ng/mL.

#### 2.5.3. Determination of IL-1β Concentration

IL-1β was measured with the Salivary IL-1β sandwich immunoassay kit (Salimetrics, State College, PA, USA) according to the manufacturer’s description (Item Number: 1-3902-5) with SPECTROstar Nano (BMG Labtech GmbH, Ostenberg, Germany). The IL-1β detection range was 3.13–200 pg/mL. The optical density (OD) was measured at 450 nm wavelength.

#### 2.5.4. Determination of TNFα Concentration

TNFα was measured with a TNFα ELISA kit (BioVendor, Laboratorní medicína a.s., Brno, Czech Republic) according to the manufacturer’s instructions (Catalogue Number: RAF128R) using SPECTROstar Nano (BMG Labtech GmbH, Ostenberg, Germany). The levels of TNFα were reported in pg/mL of saliva. The detection range was 7.8–500 pg/mL. The optical density (OD) was measured at 450 nm wavelength.

#### 2.5.5. Determination of IL-2 Concentration

IL-2 was measured with the LEGEND MAX™ Human IL-2 ELISA Kit (BioLegend, 8899 BioLegend Way, San Diego, CA 92121m USA) according to the manufacturer’s description (Product Cat. No: 431807) with SPECTROstar Nano (BMG Labtech GmbH, 77799 Ostenberg, Germany). The Il-2 detection range was 15.6–1000 pg/mL. The optical density (OD) was measured at 450 nm wavelength.

#### 2.5.6. Determination of IL-6 Concentration

IL-6 was measured with the Salivary IL-6 sandwich immunoassay kit (Salimetrics, State College, PA 16803, USA) according to the manufacturer’s description (Item Number: 1-3602-5) with SPECTROstar Nano (BMG Labtech GmbH, 77799 Ostenberg, Germany). The IL-6 detection range was 1.56–100 pg/mL. The optical density (OD) was measured at 450 nm wavelength.

#### 2.5.7. Determination of Melatonin Concentration

Melatonin was determined with a competitive immunoassay, the Melatonin Enzyme Immunoassay Kit (Salimetrics, State College, PA 16803, USA), according to the manufacturer’s description (Item Number: 1-3402-5) with SPECTROstar Nano (BMG Labtech GmbH, 77799 Ostenberg, Germany). The detection range of melatonin was 0.78–50 pg/mL. The optical density (OD) was measured at 450 nm wavelength.

### 2.6. PCR Method

#### 2.6.1. RNA Isolation with the MagMax Viral RNA Isolation Kit

RNA was isolated with the MagMax Viral RNA Isolation Kit as previously described by Yu-Hsiang, et al. [31]. The MagMax Viral RNS Isolation Kit (Applied Biosystems, Waltham, MA, USA, 1939M Rev.E) was used for RNA isolation because it is mainly suitable for virus RNA isolation [31], which inactivates nucleases at the same time [32,33]. 

#### 2.6.2. RNA Transcription

The RNA samples are diluted with distilled water for the same value (16 µL), which is calculated with a given formula in Microsoft Excel. A total of 50 ng of RNA is transcripted from every sample. The RNA and distilled water were measured into each well of the 96-well plate, and we also pipette 4 μL of dark blue LunaScript RT SuperMix. The plate is placed into conventional PCR equipment (Applied Biosystems, Thermal Cycler 2720), where the proper heat profile is set. The protocol for the RNA transcription is 25 °C for 2 min, 55 °C for 10 min, and 95 °C for 1 min. At the end of the process, the cDNA is stored at −20 °C until further usage.

The primers and probes for the genes of inflammatory factors are shown in Table 1. We mix the reagents and pipette 18.5 μL of mix and 1.5 μL of DNA into each well of the 96-well plates, then cover the plate with a transparent foil and put it into the Light Cycler 96 PCR (*Roche*) equipment. Two parallel measures were conducted from each sample and from the inner control gene (GAPDH) to avoid the errors with gene instability in our samples. We look for a stable gene with the TaqMan probe to identify the true level of IL-1β, IL-2, IL-6, and TNFα in the saliva samples. (Table 1) qPCR cycles include 95 °C at 60 s (1 cycle)*,* 95 °C at 15 s (40–45 cycles), and 60 °C at 30 s (40–45 cycles).

### 2.7. Statistical Analysis of the PCR, ELISA Results and Clinical Data

Livak method: The real-time PCR reaction was performed with cDNA from the saliva samples. The resulting C_T_ values were analyzed with the Livak method. The 2^−∆∆C^_T_ method was used to calculate the relative changes in gene expression identified with real-time qPCR [34].

The data pertaining to IL-1β (IL-2, IL-6) and TNFα inflammatory cytokines for both unstimulated and stimulated saliva were analyzed with the ANOVA method (GraphPad Prism 8). The differences were considered statistically significant if the *p* value was lower than 0.05. 

The Pearson correlation coefficient was performed with GraphPad Prism (Version 9.4.1). The results were considered statistically significant if the *p* value was <0.05.

### 2.8. Determination of Ca^2+^ Concentrations

The Ca^2+^ concentrations were determined with a Roche/Hitachi Cobas Ca^2+^ analyzer. The measurements were performed with single-lot cassettes. PreciControl ClinChem Multi 1 lot 05117003 (20 × 5 mL) PCC1 and PreciControl ClinChem Multi 2 lot 05117216 (20 × 5 mL) (Roche, Mannheim, Germany) with the reagents R1 (CAPSO(3-(cikloxeil-amino)2-hidroxi-1-propan-sulfonacid): 557 mmol/L, NM_BAPTA: 2 mmol/L, pH 10.0, non-reactive surfactant, preservative) and R2 (EDTA 7.5 mmol/L, pH 7.3, non-reactive surfactant, preservative) were used for the determination of the concentrations. The first step was preparation through a washing procedure. The second step was the calibration based on the manufacturer’s instructions with commercial calibrators, and then the analyzer measured the Ca^2+^ concentrations. After the calculations, the washing procedure was repeated. 

## 3. 16S rRNA (Svedberg Ribonucleic Acid) Gene Sequencing Method

### 3.1. Isolation of Saliva Samples 

The 10–10 saliva samples were pooled from each group by age (AG I_B, AG I_F1, AG I_F2) and by toothbrush change (BR/B, BR/F1, BR/F2, NBR/B, NBR/F1, NBR/F2, where BR represents the group who changed the toothbrush and NBR represents the group who did not change). The main steps of 16S rRNA gene sequencing were the same as in our earlier publication [27].

The mechanical lysis was started with the vortexing of the mixture containing 250 μL of saliva sample and 800 μL of CD1 Solution (Qiagen, Hilden, Germany). Incubation was performed in the supplied Bead Tubes (Qiagen, Hilden, Germany) according to the manufacturer’s protocol in the MagNa Lyser Instrument (Roche Applied Sciences, Penzberg, Germany). The vortexing and incubation steps were repeated again. After lysis, DNA isolation was performed with the commercial DNeasy PowerSoil Pro Kit (Qiagen, Hilden, Germany) according to the manufacturer’s protocol for inhibitor removal steps. The DNA concentrations were determined after the isolation with the Qubit Fluorometric Quantitation dsDNA assay kit (Thermo Fisher Scientific, Frederick, MD, USA) on a CLARIOStar microplate reader (BMG Labtech, Ortenberg, Germany). Then all of the isolated saliva samples were diluted to 1 ng/μL with PCR (polymerase chain reaction)-grade water. The Nanodrop 2000 Spectrophotometer (Thermo Fisher Scientific, Frederick, MD, USA) was used for the purity measurement of absorbance at 260 and 280 nm wavelengths at 1.7–2.0 (A260/A280) and 1.8–2.2 (A260/A230) optimal values of absorbance ratios. The DNA samples after purification were refrigerated at −20 °C.

All DNA extraction steps were performed under a class II laminar air-flow cabinet with sterile surgical gloves and face masks (for collecting samples). The negative isolation control (NIC) experiments were performed in parallel with the substitution of samples with PCR (polymerase chain reaction)-grade water. The V3–V4 PCR were conducted with eluted NIC samples. DNA-free ultraviolet (UV)-sterilized AirClean PCR workstations/cabinets were used for indexing. The 4200 Tape Station system (G2991AA; Agilent Technologies, Santa Clara, CA, USA) with Agilent D1000 ScreenTape (5067–5365) (Santa Clara, CA, USA) was used during the PCR clean-up steps of library preparation and for the validation of NIC amplicons. lllumina MiSeq paired-end (PE, 2 × 301 nt) sequencing runs with 5% PhiX spike-in quality control (PhiX Control Kit v3—FC-110-3001) were used (Illumina Inc., San Diego, CA, USA) for measurement of the overall quality [27,35].

### 3.2. Library Preparation

The Illumina 16S Metagenomic Sequencing Library Preparation Protocol (15044223 Rev. B) was used for the standard library preparation. The Illumina MiSeq benchtop sequencer was used for the sequencing of the V3 and V4 hypervariable regions of the bacterial 16S rRNA gene, generating ~460 bp amplicons by using the universal primer set: 341F-5′ CCTACGGGNGGCWGCAG 3′ and 785R-5′ GACTACHVGGGTATCTAATCC 3′ primers flanked by Illumina overhang adapter sequences (forward overhang: 5′ TCGTCGGCAGCGTCAGATGTGTATAAGAGACAG 3′, reverse overhang: 5′ GTCTCGTGGGCTCGGAGATGTGTATAAGAGACAG 3′) (Sigma Aldrich, Missouri, MO, USA).

Amplicon PCR was performed with 2 × KAPA HiFi, HotStart ReadyMix. Dual indexing of the 96 samples (i7-N7xx-12 items, i5-S5xx-8 items) was made with the Illumina Nextera XT Index Kit (FC-131-1001/2). MagSI Pure Beads (KAPA Biosystems, USA, Wilmington, Massachusetts, MA) were used for the PCR clean-ups and amplicon size selections according to the manufacturer’s instructions (KR1245—v3.16). We determined ~550–630 bp of the final libraries. PCR Agilent D1000 screen tapes (5067–5582) and D1000 Reagents (5067–5583) were used for the check-ups.

Each of our 16S amplicon libraries was measured by qPCR. In terms of amplicon sizes, they were normalized and then pooled together into one library by an equivalent molar amount. Denaturing of 5 μL of the 4 nM DNA library pool was performed with 0.2 M NaOH, followed by its dilution to a final concentration of 8 pM. The MiSeq Reagent Kit v3–618 cycle (MS-102–3003) was used for sequencing on the Illumina MiSeq platform based on the manufacturer’s protocols (Illumina Inc., San Diego, CA, USA) [27,35].

### 3.3. Bioinformatic Analysis

Phylogenetic analysis. Raw FASTQ files were downloaded and analyzed with the QIIME 2 (version: 2021.8) pipeline (URL: https://qiime2.org/, accessed on 1 August 2023). At the beginning, we imported the FASTQ files into QIIME2 format. CTGTCTCTTATACACATCT was checked to remove the remainder of the adapter sequences. Cutadapt software (in the Qiime 2 pipeline, ver. 4.7) was used for trimming from the 3′ end of the reads. We performed quality trimming in the DADA2 software with the following settings: nothing was removed from the start at both the forward and reverse reads. The length was 300 bases at the forward, while the length was set to 256 bases at the reverse [27,35,36].

Taxonomic alignment. The Naïve Bayesian machine learning-based classifier was used for taxonomic alignment with the Human Oral Microbiome Database (HOMD, http://homd.org/, ver. 15.23, accessed on 1 August 2023). The align-to-tree-mafft-FastTree plugin, which is integrated in the QIIME 2 pipeline, was used for the calculation of phylogenetic trees [27,35].

Statistical analysis. To analyze ’alpha diversity,’ the Chao1, Faith, Shannon, and Simpson indexes were calculated in the QIIME 2 pipeline. The significance of the differences was measured with the Kruskal-Wallis pairwise test [27,35].

Data visualization. The calculation and visualization of the heat-tree matrix were made with the Metacoder package in R. The differences between groups (B (baseline), AC S (Anthocyanin Stimulated Saliva), AC US (Anthocyanin Unstimulated Saliva), BR, NBR) were shown on heat-trees by the log_2_ median ratio proportions and operational taxonomic unit (OTU) counts.

The ’Wilcox rank-sum test’ was used for the calculation of statistical differences in heat-trees. The construction of figures was made with ’ggplot2 (ver. 2.3.4.3), an R *package*’ [27,35].

### 3.4. Core Microbiome

To determine the effects of AC gum chewing, core microbiomes were considered using OTUs represented in 100% of all saliva samples. Comparisons in the core bacterial genera scattering plot were made related to their relative frequency distributions.

### 3.5. Correlation between the Microbiota, Cytokines, Mucins, and Melatonin

We performed cluster analysis in relation to the bacterial genera with the relative gene expression and protein concentrations of mucins, cytokines, and melatonin using Pearson correlation. For cluster heatmap visualization, the Seaborn Python package was used.

## 4. Results

### 4.1. DMF-T Status of the Groups by Toothbrush Change and Age

We performed our investigation on the same cohort as in our earlier publication [27] with the same features (group compositions by age and toothbrush change, DMF-T, mean age). The average BPE (basic periodontal examination) index was 0.32 in the BR group, while it was 0.53 in the NBR group. There was no statistically significant difference between the two groups with ANOVA in the DMF-T and BPE status, but the age between the two age groups was differed significantly (*p* = 0.0003).

Table 2 shows the clinical status of the age groups.

**Figure 2:**
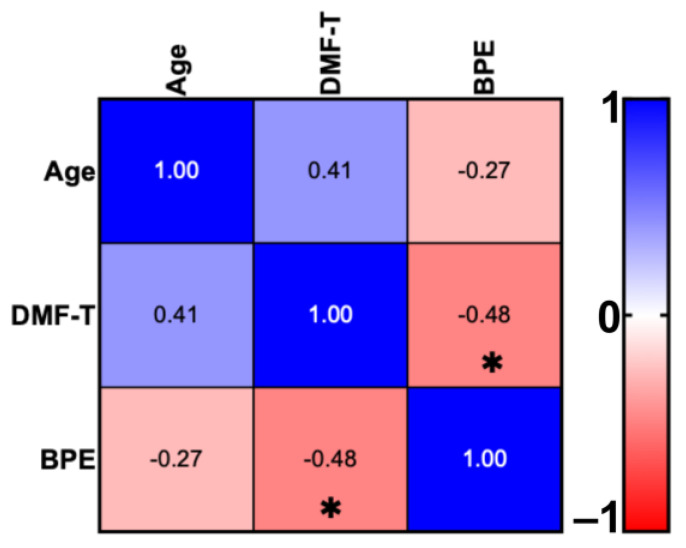
Pearson correlation between the age, DMF-T (number of decayed, missing, and filled teeth), and BPE (basic periodontal examination) indexes. * *p* = 0.0332.

The Pearson correlation showed a significant negative correlation (−0.48) between the DMF-T (6.9 ± 4.97) and BPE (0.42 ± 0.34) of the participants (Figure 2).

We could not completely exclude gingival inflammation (participants with a BPE index > 0) as we included participants in our experiment with similar geographical and living conditions. This was influenced by factors such as oral hygiene and dietary habits.

### 4.2. Pro-Inflammatory Cytokines, Mucins, and Melatonin Levels in Unstimulated and Stimulated Saliva

#### 4.2.1. Comparison of Unstimulated and Stimulated Saliva

The results of the investigated parameters in unstimulated and stimulated saliva showed that the level of MUC5B did not change significantly, neither during the investigational period nor upon stimulation, but there was a significance between unstimulated and stimulated saliva. Otherwise, the level of MUC7 was significantly higher in the stimulated saliva at almost every sampling occasion compared to the unstimulated saliva.

The mRNA expression of IL-1β did not show any statistically significant difference during the course of AC treatment both in stimulated and unstimulated saliva, but its expression was significantly higher in stimulated saliva. It is unsurprising that the protein concentration of IL-1β changed in the opposite way. TNFα mRNA concentration was significantly higher on Day 10 (1.91) in the stimulated saliva. TNFα protein expression was significantly higher in the stimulated saliva samples.

It is interesting that we did not observe IL-2 and IL-6 mRNA in the unstimulated and stimulated saliva samples. The protein concentration of IL-2 showed a not-significant decrease in unstimulated and stimulated saliva, while the level of IL-6 in the stimulated saliva was significantly lower than the Day 10 of unstimulated saliva except on the last day of the experiment.

The calcium levels showed a significant difference in stimulated saliva between the control and Day 11 (*p* = 0.0002) and Day 21 of sampling (*p* < 0.0001).

**Figure 3:**
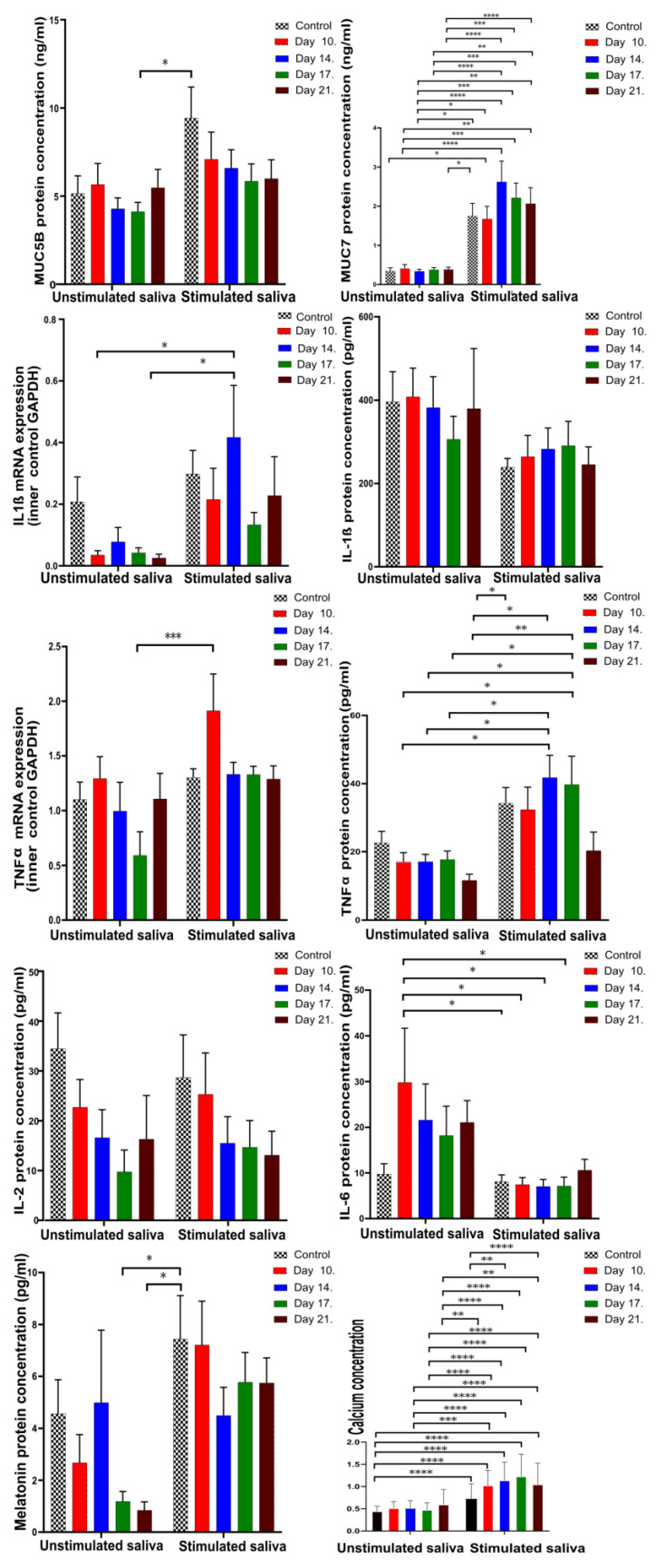
Mucins (MUC5B, MUC7), inflammatory marker (IL-2, IL-6, IL-1β, TNFα) mRNAs and protein concentrations, and the amount of melatonin and Ca^2+^ in unstimulated and stimulated saliva (* *p* = 0.0332, ** *p* = 0.0021, *** *p* = 0.0002, and **** *p* < 0.0001). Black and white squared: control. Sampling occasions during AC treatment: red—10th day, blue—14th day, green—17th day, and brown—21st day.

In stimulated saliva, the MUC5B, IL-1β mRNA, TNFα, TNFα mRNA, melatonin, MUC7, and Ca^2+^ levels were significantly higher, with the exception of IL-1β protein and IL-2, while IL-6 was significantly higher in the unstimulated saliva (Figure 3, Table 3).

#### 4.2.2. The Effects of Toothbrush Change on Unstimulated Saliva

In unstimulated saliva, we observed a significant difference in IL-1β between the BR and NBR groups by toothbrush change, where the tendency was opposite: in the case of the BR group, it was reduced, while in the case of the NBR group, it was elevated until the end of the investigation. The IL-1β mRNA expression also showed significant differences between the two groups, as indicated by the values of the BR group; otherwise, until the end of the experiment, it was reduced in both groups. As the IL-1β mRNA was also significantly reduced for the Day 10 (*p* = 0.0332) we can propose that the mRNA was completely used for protein synthesis.

We observed further significant differences in the values for TNFα and MUC7 during the first sampling of the chewing gum with anthocyanin. MUC7 and IL-1β showed similar, but not significant, differences: after the control period, they decreased in the BR group but increased in the NBR group until the end of the study.

**Figure 4:**
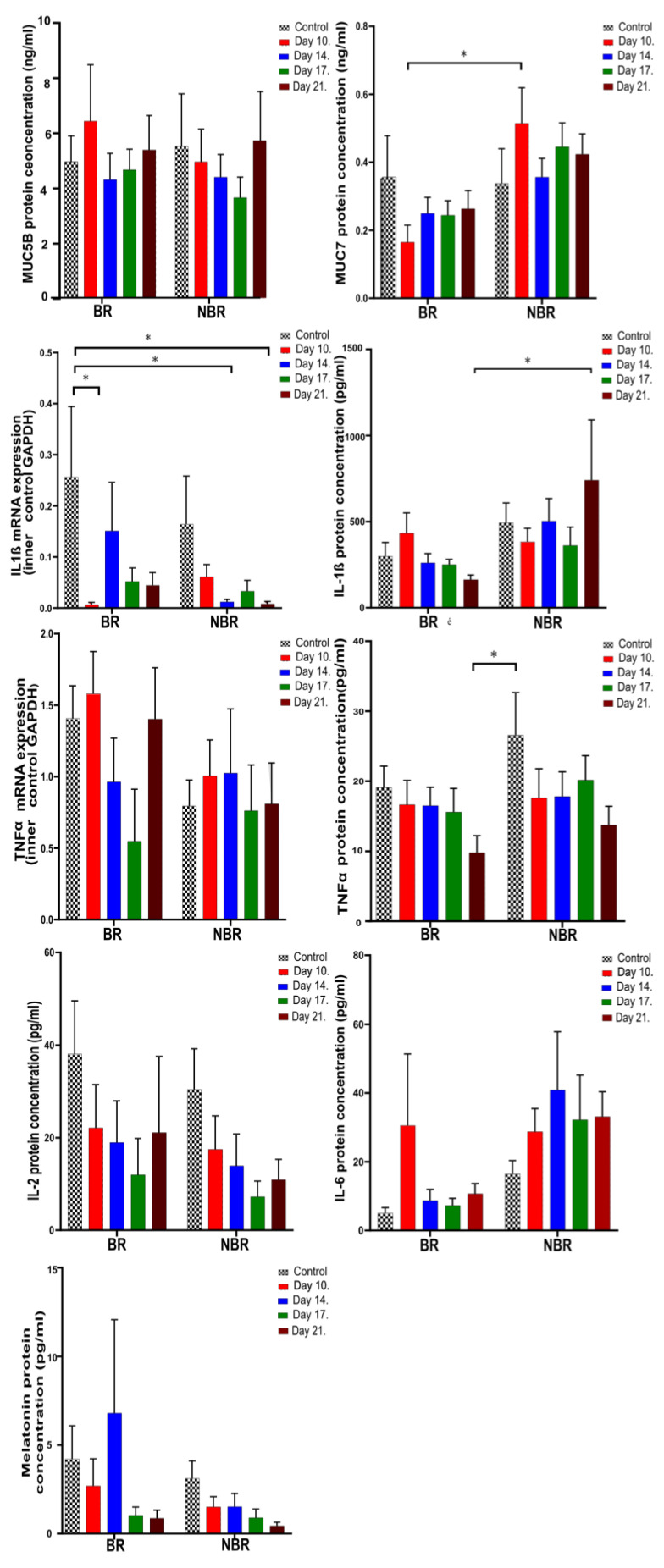
Mucins (MUC5B, MUC7), inflammatory marker (IL-1β, TNFα, IL-2, IL-6) mRNA and protein concentrations, and melatonin concentrations in unstimulated saliva (* *p* = 0.0332).

In relation to the toothbrush change, we did not observe any significant difference in the TNFα mRNA expression or the MUC5B, IL-2, and melatonin protein concentrations. During the study, the IL-2 and melatonin levels showed a similar, but not significant, tendency: the levels were reduced in both groups until the end of the study, but in the case of IL-6, we observed a different tendency: it was elevated until the end of the study (Figure 4). 

**Figure 5:**
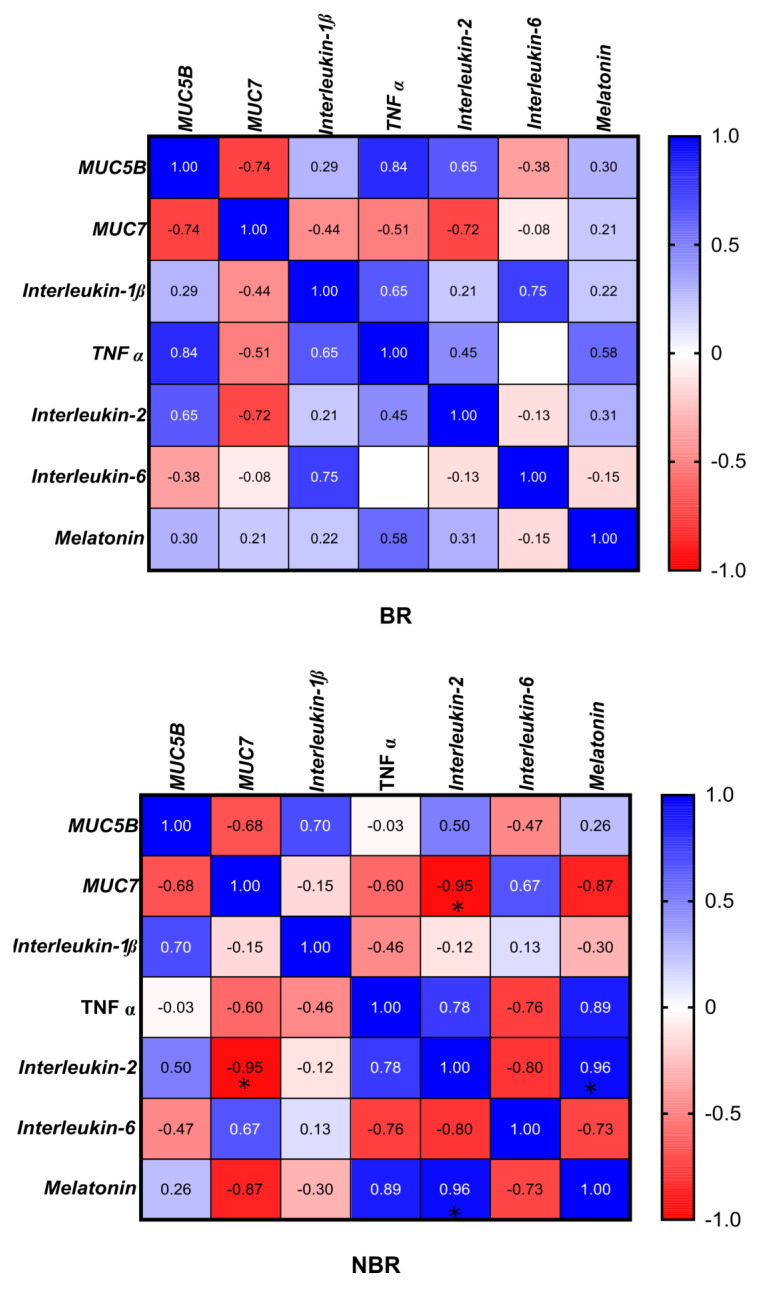
Pearson correlation between cytokines (IL-2, IL-6, IL-1β, TNFα), mucins (MUC5B, MUC7), and melatonin in unstimulated saliva. * *p* = 0.0332. BR: The index in the BR group. NBR The index in the NBR group.

The results of the Pearson correlation showed a significant negative correlation (−0.95) between IL-2 and MUC7 in the case of the NBR group. Further significance could be seen in this group between IL-2 and melatonin (0.96) (Figure 5).

#### 4.2.3. The Effects of Toothbrush Change on Stimulated Saliva

The MUC5B in the BR group showed a decreasing tendency, but it was always higher than the values of the NBR group. We observed further significance between Day 10 of the BR group (10.6) and Day 10 (3.65) and Day 17 (2.9) of the NBR group. 

**Figure 6:**
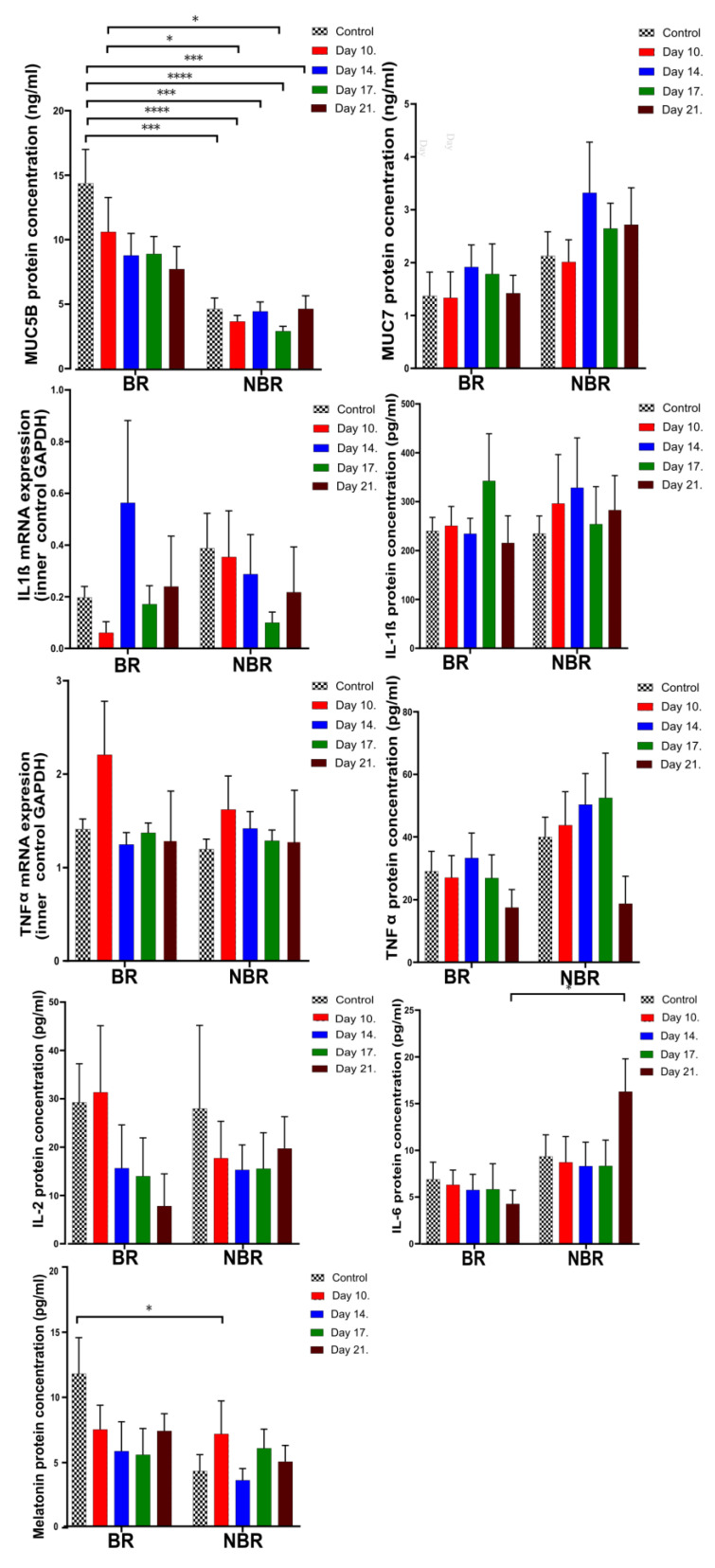
Mucins (MUC5B, MUC7), inflammatory markers (IL-2, IL-6, IL-1β, TNFα), melatonin mRNA, and protein concentrations in stimulated saliva (* *p* = 0.0332, *** *p* = 0.0002, **** *p* < 0.0001).

We saw similar results in the values of melatonin, where the control of the BR group (11.7) was significantly higher than the value on Day 10 (7.1) of the NBR group. In relation to the further sampling appointments, we saw similar tendencies as in the case of MUC5B, but in the case of melatonin, the values in the NBR group were elevated until the end of the investigation (4.96) in comparison with the control values (4.24). Further significant differences were observed in the case of IL-6, where the values of the BR group were generally lower than in the NBR group. In the former group, the value was reduced (4.27), while in the latter, it was elevated (16.28) until the end of the investigation (Figure 6).

**Figure 7:**
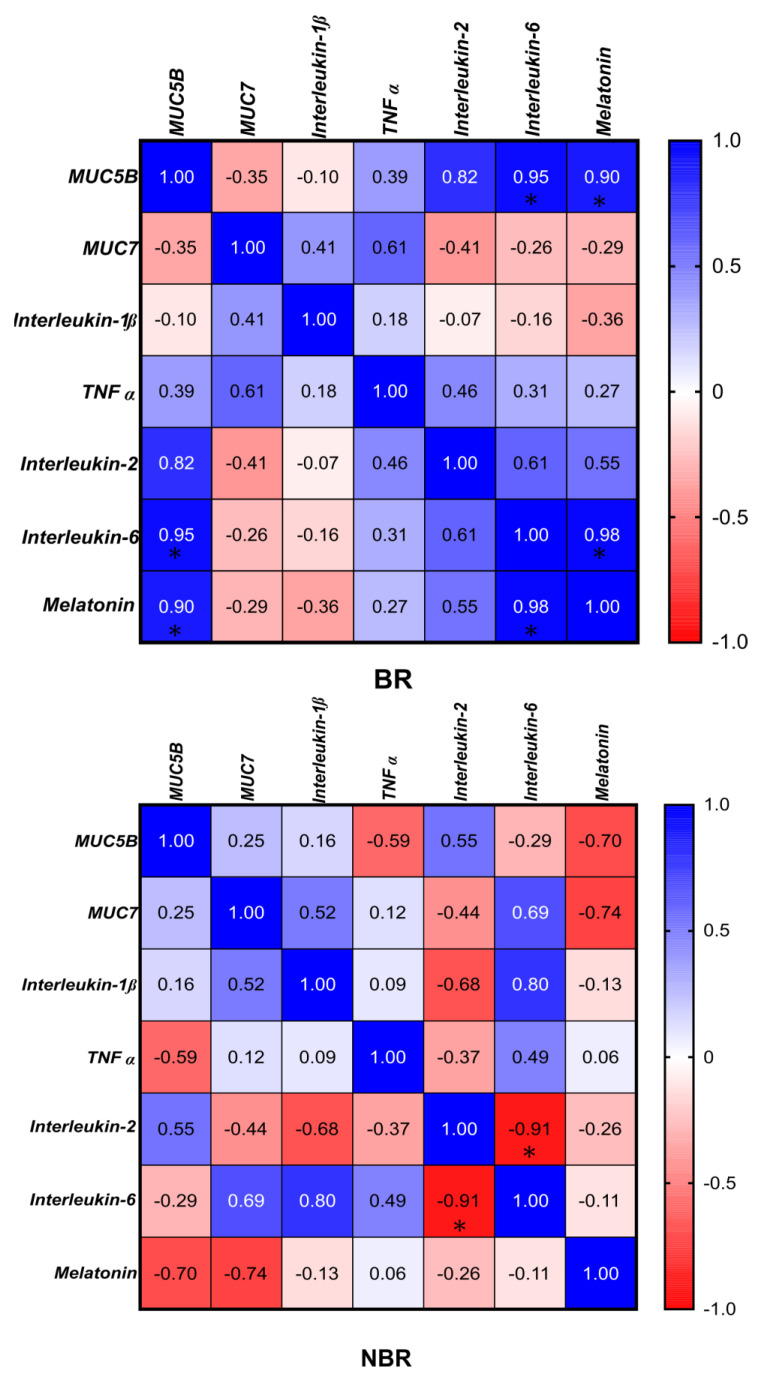
Pearson correlation between cytokines (IL-2, IL-6, IL-1β, TNFα), mucins (MUC5B, MUC7), and melatonin in stimulated saliva. * *p* = 0.0332. BR: he index in the BR group. NBR: The index in the NBR group.

We did not observe any significant difference between the groups by toothbrush change in the MUC7, IL-1β, and TNFα mRNA and protein concentrations. We also did not observe differences in the unstimulated or stimulated Ca^2+^ concentrations. As shown by the Pearson correlation, in the BR group, the IL-6 and MUC5B (0.95), IL-6 and melatonin (0.98), and MUC5B and melatonin (0.9) were positively correlated with each other, while in the NBR group, the IL-2 and IL-6 were significantly negatively (−0.91) correlated with each other (Figure 7).

#### 4.2.4. Alpha Diversity

##### Comparison of Unstimulated and Stimulated Saliva

**Figure 8:**
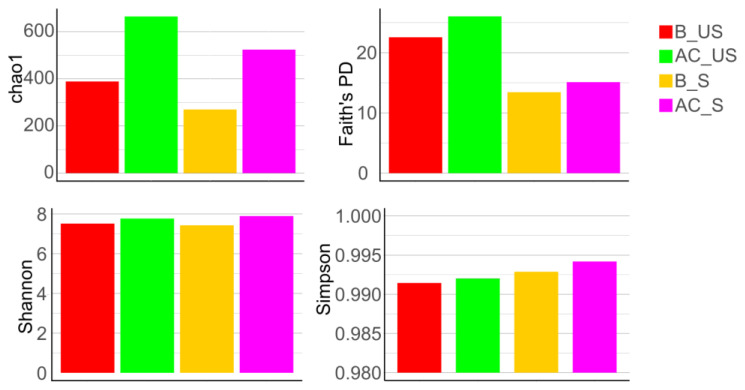
Alpha diversities (Chao1, Faith’s PD, Shannon, and Simpson) in unstimulated and stimulated saliva. The color codes of groups are: red: B_US—baseline unstimulated; green: AC_US—anthocyanin unstimulated; yellow: B_S—baseline stimulated; purple: AC_S—anthocyanin stimulated.

When we compared alpha diversities (Chao1, Faith’s PD, Shannon, and Simpson) in the unstimulated and stimulated saliva, we found greater values in the unstimulated saliva (B_US, AC_US) compared to the stimulated saliva (B_S, AC_S). During the course of AC treatment (AC_US, AC_S), the diversity was greater than in the baseline period (B_US, B_S) (Figure 8).

##### Alpha Diversity of Saliva in Groups Based on Toothbrush Change

**Figure 9:**
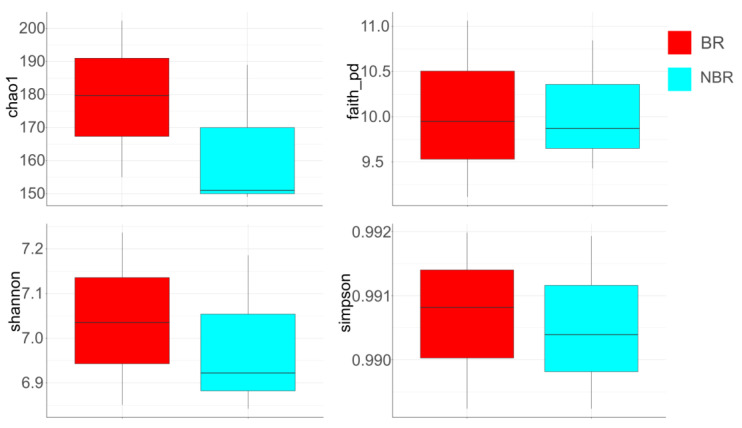
Alpha diversities (Chao1, Faith’s PD, Shannon, and Simpson) of the pooled saliva samples in relation to the groups by toothbrush change. The color codes of groups are: BR—red, NBR—blue.

The *Chao1* and *Shannon* diversities showed that the NBR group had a lower diversity than the BR group, but these results were not significant. Regarding the *Faith’s PD* and *Simpson* diversities, minor differences were observed between the two groups by toothbrush change, but the tendency was the same (Figure 9).

#### 4.2.5. Heat-Tree of Microbiota Profiles in Saliva

##### Heat-Tree Demonstrating Unstimulated and Stimulated Saliva

**Figure 10:**
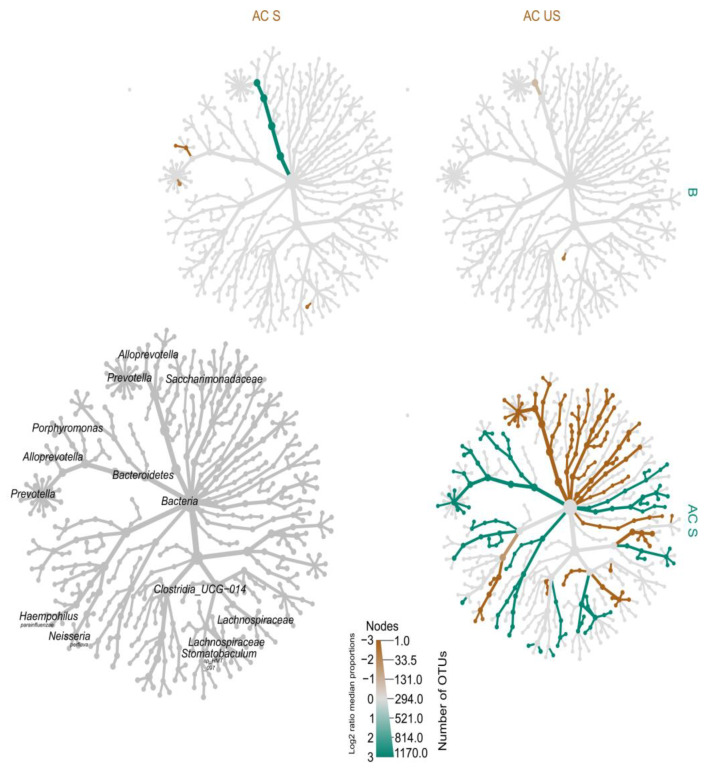
Heat-tree of the log_2_ ratio median proportion of B (baseline), AC_US (anthocyanin unstimulated), and AC_S (anthocyanin stimulated).

Metacoder analysis showed a significantly higher log_2_ median proportion of *s_Prevotella_melaninogenica* (−2.175) and *s_Neisseria_perflava* (−2.693) in AC_US (unstimulated) as compared to AC_S (stimulated). It was also higher in the cases of *f_Clostridia_UCG_014* (−3.309), *f_Lachnospiraceae* (−5.109), *f_Prevotellaceae* (−3.823), *f_Neisseriaceae* (−3.485), the *genera Prevotella* (−3.616), and *Neisseria* (−3.429). In the case of stimulated saliva, we detected only *Stomatobaculum sp_HMT_097* (−2.739) and *genus Alloprevotella* (−4.223) in a higher log_2_ median proportion as compared to the B (baseline) (Figure 10).

##### Heat-Tree Demonstrating Stimulated Saliva

The BR group was characterized by a higher log_2_ ratio of *Porphyromonas* (*Porphyromonas endodontalis* (1.004) and *Porphyromonas pasteri* (0.821)), *Capnocytophaga leadbetteri* (1.94), and the family of *Fusobacteriaceae* (0.799).

**Figure 11:**
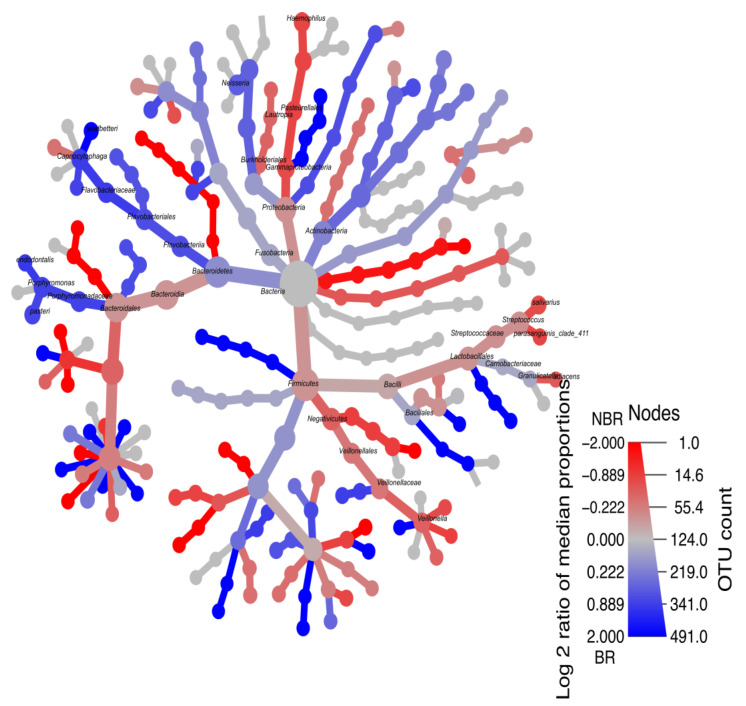
Heat-tree of the stimulated saliva (BR—blue, NBR—red).

The NBR group was represented by a higher log_2_ ratio of mainly *Streptococcus* (*Streptococcus salivarius* (−0.707) and *Streptococcus parasanguinis_clade_411* (−0.805)), *Haemophilus parainfluenzae* (−0.789), and *Granulicatella adiacens* (−0.567) (Figure 11).

#### 4.2.6. Core Microbiome

**Figure 12:**
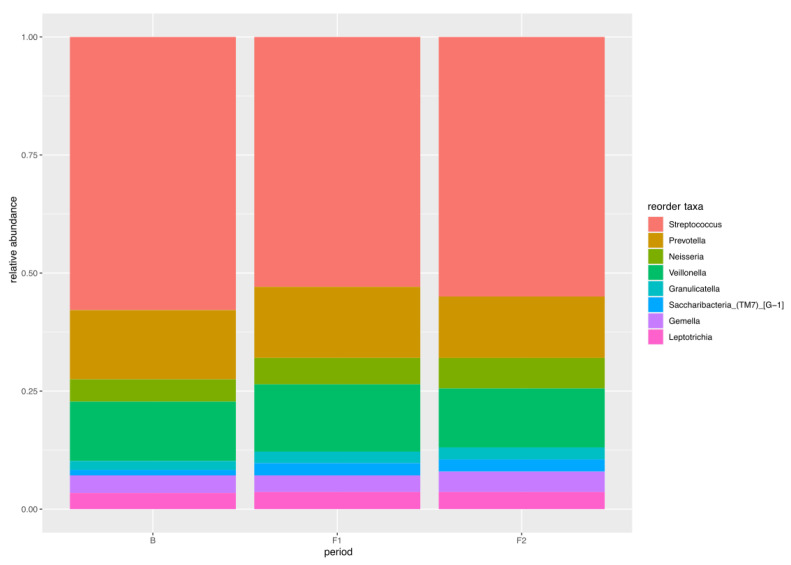
The relative frequency distribution of the core microbiome taxa (Streptococcus, Prevotella, Neisseria, Veillonella, Granulicatella, Saccharibacteria_(TM7)_[G_1], Gemella, and Leptotrichia) in stimulated saliva based on the treatment periods (B, F1, and F2).

The 100% core oral microbiota between the participants according to the average of their relative frequency were *Streptococcus* (0.55), *Prevotella* (0.14), *Veillonella* (0.13), *Neisseria* (0.056), *Granulicatella* (0.023), *Saccharibacteria_(TM7)_[G_1]* (0.021), *Gemella* (0.039), and *Leptotrichia* (0.036) (Figure 12).

#### 4.2.7. Correlation of the Microbiota with Cytokines, Mucins, and Melatonin

We observed opposite tendencies in relation to MUC5B and MUC7 in the cases of the genera: Actinomyces (MUC5B: 0.258, MUC7: −0.462), Lachnospiraceae_[G-3] (MUC5B: 0.775, MUC7: −0.644), Eikenella (MUC5B: 0.658, MUC7: −0.301), Capnocytophaga (MUC5B: 0.250, MUC7: −0.362), Porphyromonas (MUC5B: 0.415, MUC7: −0.288), Peptrostreptococcae_[G-9] (MUC5B: 0.507, MUC7: −0.431), Alloprevotellae (MUC5B: −0.537, MUC7: 0.308), Bacteriodales_[G-2] (MUC5B: 0.424, MUC7: −0.19), Veillonella (MUC5B: −0.428; MUC7: 0.476), Bifidobacterium (MUC5B: −0.284, MUC7: 0.36), Haemophilus (MUC5B: −0.641, MUC7: 0.615), Bacteriodetes_[G-5] (MUC5B: −0.481, MUC7: 0.517), Mogibacterium (MUC5B: −0.374, MUC7: 0.242), Ruminococcae_[G-1] (MUC5B: −0.1; MUC7: 0.32), and Peptococcus (MUC5B: −0.311, MUC7: 0.407) (Table 4).

In addition, IL-6 had a positive correlation with *Haemophilus* (0.518). The MUC5B had a positive correlation (0.424), and the MUC7 had a negative correlation (−0.19) with the *genus Bacteroidales_[G−2].* The MUC7 had a positive correlation (0.517), while the MUC5B had a negative correlation (−0.481) with the *genus Bacteroidetes_[G−5].* IL-1β had a positive correlation (0.472) with the latter one. The MUC7 showed further positive correlation with the *genus Veillonella* (−0.476). The MUC5B also had a positive correlation with the *genus Streptococcus* (0.166).

The MUC5B and melatonin both showed positive correlations with the *genera Lachnospiraceae_[G−2]* (MUC5B: 0.168, melatonin: 0.021), *Eikenella* (MUC5B: 0.658, melatonin: 0.744), and *Saccharibacteria_(TM7)_[G−5]* (MUC5B: 0.658, melatonin: 0.743).

The melatonin and TNFα both had positive correlations with *genus Leptotrichia* (melatonin: 0.520, TNFα: 0.479). The MUC5B had a negative correlation (−0.476), while the IL-1β (0.535) and IL-6 (0.396) were positively correlated with the *Granulicatella*. 

**Figure 13:**
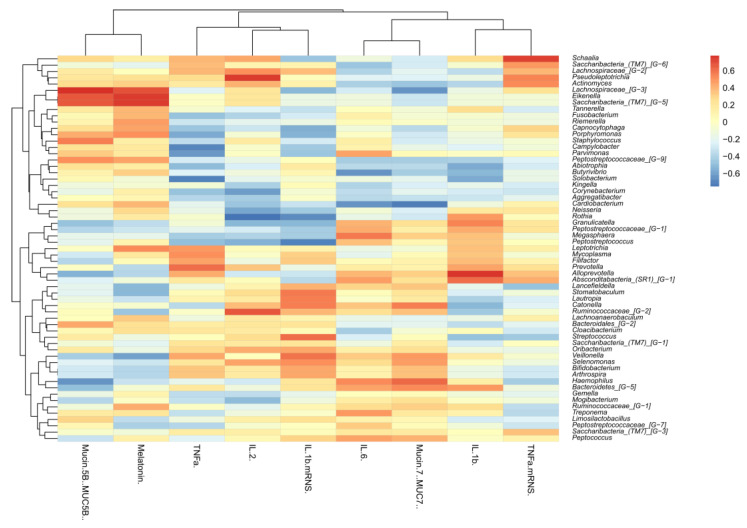
Correlation of mucins, melatonin, and cytokines with the salivary microbiota.

The *genus Cardiobacterium* correlated negatively with the MUC7 (−0.718) and the IL-6 (−0.63). The TNFα correlated negatively with the *genera Porphyromonas* (−0.569) and positively with the *Prevotella* (0.607). The IL-6 showed a smaller but positive correlation (0.162) with the latter one. The IL-2 showed a positive correlation with the *genus Schaalia* (0.432) and *Actinomyces* (0.400), while it had a negative correlation with the *genus Neisseria* (−0.579) and *genus Rothia* (−0.752). The IL-1β had a positive correlation (0.475) with the latter one. The IL-1β had a negative correlation with the *genus Streptococcus* (−0.463), while on the other hand, it had a positive correlation with its mRNA (0.603) (Figure 13).

## 5. Discussion

In our previous study, we investigated the effects of preventive interventions on unstimulated salivary microbiota, including a chemical intervention: chewing an AC-containing gum, and a physical intervention: scaling over the course of mechanical plaque control, followed by a toothbrush change or not. We found a reduced level as the effect of toothbrush change in the case of mainly Gram-negative anaerobes (*Prevotella melaninogenica, Porphyromonas pasteri,* and *Fusobacterium nucleatum subsp. vincentii*) and a Gram-positive coccus (*Rothia mucilaginosa*) [27]. The anthocyanins competitively inhibit the activity of α-amylase as well as the attachment of *S. mutans* to tooth surfaces [25]. Additionally, in the present study, we investigated the action of anthocyanins on mucins, pro-inflammatory cytokines, melatonin, Ca^2+^ levels, and the oral microbiome. Furthermore, we mapped the possible interactions between these factors and the oral microbiome and characterized the stimulated salivary microbiota. Hence, aim of the present study was to investigate the microbial ecosystem in saliva and how it maintains the symbiosis with natural chemical products [37], reported not to be harmful for the physiological processes of the body in a healthy population [38].

The current evidence in the literature suggests that MUC5B and MUC7 can be differentiated by their functions [39]. We obtained similar results on the level of mucins in unstimulated and stimulated saliva [39]. MUC5B is responsible for the viscosity of saliva and binds to the hydroxyapatite on the enamel, while MUC7 indirectly determines the elasticity of saliva [40,41]. These differences can be related to their structural differences, as MUC5B is the main gel-forming protein of the saliva, an oligomeric glycoprotein > 1000 kDa, and MUC7 is a low-weight (120–150 kDa) single glycoprotein with a monomeric structure [42]. For this reason, MUC5B can selectively bind microorganisms (e.g., *Haemophilus parainfluenzae*) [19], while MUC7 can bind them directly by their dispersion [43]. A further difference between the two mucins shows that MUC5B is able to keep *S. mutans* in a planktonic form due to its structure [13]. These data were supported by the results of our microbiome correlation, as the level of MUC5B was positively correlated with the level of genus *Streptococcus* (0.166) (Figure 13).

During the course of AC treatment in stimulated saliva, continuously decreasing concentrations of MUC5B (B: 9.477, F2 (Day 21): 7.721) were observed after the scaling (Figure 3), and the toothbrush change promoted and acted as a probiotic that maintained the formation of a healthy oral microbiota. This can be explained by the investigations of Wickström, et al. [44], who found that bacteria in the diet can cooperate with the induction/up-regulation of MUC5B-degrading enzymes. Hence, the bacterial endopeptidase activity led to the first step of MU5B degradation, which provides further protection for a healthy microbiome [44].

The levels of MUC7 were significantly higher in the NBR group in the unstimulated saliva, while the levels of MUC5B in the stimulated saliva in the BR group had a higher DMF-T, which was in line with the MUC5B and MUC7 levels detected in the investigation conducted by Gabryel-Porowska, et al. [45].

In the background of dental caries, the effects of several cytokines have been hypothesized, in which IL-1β and TNFα play a central role [46], because their up-regulation was demonstrated [47] in the odontoblast layer on the surface of carious teeth by in vitro [48] and in vivo experiments. Certain pro-inflammatory cytokines (e.g., TNFα) can also increase the expression of Toll-Like Receptors (TLR), hence the expression of IL-6 in the salivary glands [49], which is the central mediator of the transition from the innate immune response to the acquired immune response [17].

Based on the literature, TNFα and IL-6 are significantly increased in the saliva samples of periodontally healthy patients with high caries prevalence, so an elevated level of these factors shows an ongoing caries process or susceptibility to caries [17]. A decrease in IL-6 levels has also been reported as a result of caries treatment [18]. TNFα in unstimulated saliva and IL-6 in stimulated saliva were significantly higher in the NBR group than in the BR group, which may also indicate the continuous existence of inflammation without changing toothbrushes.

It Is also known from the literature that the MUC5B-encoding genes of MUC5AC can be up-regulated by IL-1β, TNFα, and IL-6, which also correlates positively with MUC5B in our studies [14]. This supports the positive correlation (0.95) observed between MUC5B and IL-6 in the stimulated saliva of toothbrush changers (Figure 7).

Comparing unstimulated and stimulated saliva (Figure 3), IL-6 was significantly higher in unstimulated saliva, while TNFα was significantly higher in stimulated saliva, similar to MUC5B, MUC7, melatonin, and IL-1β mRNA expression. Based on the previous literature [16], significantly higher IL-1β and TNFα concentrations in unstimulated saliva in the NBR group may be connected to background increases in IL-6 production [49]. Initially decreasing TNFα levels can eventually increase the production of MUC7. The decreasing TNFα concentration parallel to the elevated IL-6 confirms the TNFα inhibitory effect of IL-6 [17]. Biró [50] found that the AC extract induced a significant decrease in IL-6 and TNFα. Nemes, et al. [51] also reported a decrease in the expression of IL-6 and TNFα by ACs [51]. The effect of chewing AC gum can be connected to the background decreases in TNFα in both unstimulated and stimulated saliva and the decreased IL-6 levels observed in the BR group in the stimulated saliva by the end of the study. The effects observed when comparing unstimulated and stimulated saliva clearly indicate the effects of the AC.

In unstimulated saliva, the level of IL-1β was significantly higher in the NBR group (Figure 4), which can be explained by the fact that the toothbrush microbiome without a toothbrush change can continuously maintain inflammation.

Otherwise, in the stimulated saliva, the significant difference observed in IL-6 levels between the two groups by toothbrush change (Figure 6) and as the study progressed was a decrease in the BR group similar to gingivitis patients [52], in whom IL-1β and IL-6 increased during the treatment to reach the values of healthy persons.

The higher melatonin and lower TNFα levels detected in the unstimulated and stimulated saliva of the toothbrush changers compared to the group that did not change their toothbrush support the beneficial effects of changing the toothbrush after scaling on oral hygiene.

Examining the level of melatonin in caries and caries-free individuals, it was shown that higher concentrations are present in caries-free individuals [20]. The elevated melatonin level inhibits TNFα, which is clearly supported by our results in unstimulated and stimulated saliva in different groups by toothbrush change [21]. Higher melatonin levels and lower TNFα levels were measured in the BR group compared to the NBR group, which supports the beneficial action of toothbrush change on oral hygiene.

The interaction of MUC5B and MUC7 with melatonin and cytokines also acts in a different way. In our studies, MUC5B and melatonin levels were positively correlated (0.9) in stimulated saliva (Figure 7), and a significant difference was shown between the groups by toothbrush change.

In the study conducted by Dawes and Dong [24], by measuring the Ca^2+^ concentrations in unstimulated saliva and saliva stimulated by chewing sugar-free gum, it was established that the supersaturation of saliva is higher in stimulated saliva, especially with regard to hydroxyapatite. The resulting supersaturation leads to an increase in the Ca^2+^ concentration. Stimulated saliva also has a higher anticariogenic potential, which is also consistent with our studies, where we measured significantly higher levels of MUC5B, MUC7, TNFα, and melatonin in the stimulated saliva (Figure 3, Table 3).

The alpha diversity was generally lower in stimulated saliva than in unstimulated saliva (Figure 8). As a result of AC treatment, the value of alpha diversity was elevated both in the unstimulated and stimulated saliva. The latter is in line with our earlier investigation about the analysis of unstimulated saliva, where we found an initial increase during the first week of chewing the AC gum, but it then decreased while remaining higher than the baseline until the end of the investigation [27]. These results are in line with Schoilew, et al. [53], who found that the susceptibility to caries is inversely proportional to the alpha diversity. Hence, a diverse and stable microbiota shows a reduced susceptibility to caries. 

We did not detect any significant change between the two groups in terms of DMF-T status, although the control (BR) group had a slightly higher DMF-T index (8.11 ± 4.64) than the NBR (5.22 ± 4.5) group. The number of decayed teeth was practically the same in both groups (BR: 0.67 ± 0.9, NBR: 0.67 ± 1.07). The number of restored and missing teeth was higher in the BR group, so we can conclude that chewing AC gum made the composition of the oral microbiota more diverse in stimulated saliva based on *Chao1* diversity (Figure 9), which is also in line with our previous results shown in unstimulated saliva [27,53]. *Shannon diversity* also supported these results. In the case of *Faith diversity*, there was a smaller difference in the median values of the two groups, but based on our results, we can assume a more phylogenetically diverse composition of the NBR group [37,53]. *Simpson diversity* also showed minimal differences between the median values of the two groups. Despite these results, the more diverse composition of the NBR group and the presumably more acidogenic flora (more missing and filled teeth) during the AC treatment resulted in increased diversity and, thus, a more diverse and healthy microbiota composition [37,53].

Based on the significant results of the heat-tree comparing unstimulated and stimulated saliva (Figure 10), *P. melaninogenica*, which showed a decreasing level in unstimulated saliva during the previous analysis of resting saliva as a result of AC treatment, and in accordance with the literature data [8,27,54] on the characteristic of good oral hygiene, *s_Neisseria_perflava* (−2.693), *f_Clostridia_UCG_014* (−3.309), *f_Lachnospiraceae* (−5.109), *f_Prevotellaceae* (−3.828), *f_Neisseriaceae* (−3.485), and *genus Neisseria* (−3.429) had higher log_2_ ratios. In stimulated saliva, the recently discovered *Stomatobaculum sp_HMT_097* (−2.739), which we found in unstimulated saliva in our earlier study [27], and *genus Alloprevotella* (−4.223), which can also be detected in the core microbiome of Romanian and Swedish adolescents [55], were detected in stimulated saliva with a higher log_2_ ratio than the B (baseline) group.

We found that the BR group in unstimulated saliva is mainly composed of *Haemophilus, Neisseria*, and *Veilonella.* Baker, et al. [56], who investigated the association/correlation of inflammatory biomarkers and microbiota sequencing, found *Rothia, Neisseria,* and *Haemophilus* species to be connected to healthy oral hygiene. In contrast, Li, et al. [57] found a higher abundance of these species in the case of patients with caries. In line with the investigations of Simon-Soro and Ren (10), the BR group in unstimulated saliva consists of early colonizers, *Haemophilus, Neisseria,* and *Veillonella*, while the BR group in stimulated saliva (Figure 11) consists of mainly late colonizers, *Prevotella, Porphyromonas,* and *Fusobacterium*. Together, they form a microbial unit aggregated in saliva, from which the plaque is gradually formed.

In stimulated saliva in the NBR group, the presence of *Streptococcus,* which is clearly associated with caries, can be related to an increased susceptibility to caries, in accordance with our previous studies [27,58]. In the BR group, *Fusobacteriaceae* was present in both unstimulated and stimulated saliva (Figure 11). The reduced level of potential late colonizing biofilm was observed in our previous study during the analysis of unstimulated saliva samples, which is in line with the results of Miller, et al. [59], who found that chewing gum containing blackberry powder and xylitol showed a reduced level, which we attribute to the beneficial effect of AC added to xylitol-containing chewing gum [27].

In the study by Wirth, et al. [60], investigating healthy participants and patients with gingivitis, they found *Streptococcus parasanguinis* and *Streptococcus salivarius (S. salivarius)* mainly in gingivitis patients. These results were in line with our study, where we found these bacteria with a higher abundance in the NBR group. Contradictory findings state that *S. salivarius* has an anti-inflammatory effect in the oral cavity [61], yet it also occurs in dental caries [58].

In the present study, the *Streptococcus genus*, which showed the highest relative frequency (average 0.55) (Figure 12) in the core microbiome, was primarily composed of non-pathogenic species, as indicated in previously mentioned data. IL-1β was also positively correlated with *Granulicatella* (0.535) and Rothia (0.475) [56], which are associated with good oral hygiene based on our previous studies [56].

For the further analysis of the two main secreted mucins observed in our studies, Pearson’s correlation was performed to examine the relationships between bacterial genera and factors (mucins, cytokines, and melatonin) (Figure 13, Table 4). The interaction between the mucins and bacteria also allows for an interaction with pathogenic, commensal, and probiotic bacteria, which can utilize mucins through the degradation of O-glycans found in their structure [6].

The complex interaction of mucins and bacteria influences the oral microbiota’s diversity [44]. We know that we can increase the degradation of mucins by increasing bacterial counts, which provides further substrate for growth and catabolic processes (e.g., in the case of *Veilonella dispar*) [62]. In the unstimulated saliva in the BR group, we observed a higher proportion of *H. parainfluenzae* and *Veillonella rogosae* [27], where the pathogenic microflora did not recolonize the oral cavity and the AC treatment maintained the healthy microbiota [27]. In our present investigation, we found *Haemophilus* in greater proportion in the BR group, and it was correlated negatively with MUC5B (-0.641) and positively with MUC7 (0.615). In the literature, there are contradictory findings about the role of *H. parainfluenzae* in caries [56,63]. In unstimulated saliva, we found the opposite tendency, with a greater log_2_ median proportion of *H. parainfluenzae*. In the present study, the third most frequent family of the core microbiome, *Veilonella* (average 0.13) (Figure 12), was correlated positively with MUC7 (0.476) and negatively with MUC5B (−0.428).

By adjusting to the systemic mucins, the bacteria are able to degrade the histo-blood-group antigens (oligosaccharides) on them [64]. In summary, we call these mucin-degrading enzymes ‘mucinases’ [6,65].

Derrien et al. [6] found that *Bacteriodetes* have a whole set of ‘mucinase’ enzymes providing their degradation. The *genus Bacteriodales_[G-2]* was correlated positively with MUC5B (0.424) and negatively with MUC7 (−0.19). In contrast, *genus Bacteriodales_[G-5]* was correlated positively with MUC7 (0.517) [6].

The *genus Ruminococcae_[G-2]* (0.31) and *genus Ruminococcae_[G-1]* (0.32) had a smaller but still positive correlation with MUC7. These genera are able to use the mucins as a carbon source; however, the existence of a complex microbial community is important for their complete utilization [6]. Based on the literature, the *genus Bifidobacterium* also contains the whole set of mucine-degrading enzymes and is correlated positively with MUC7 [6]. The *Actinomyces genus* correlated negatively with MUC7 (−0.462) and IL-6 (−0.415), but positively with IL-2 (0.400), which strengthens the pathogenic role of this genus [66].The significant negative correlation between IL-2 and IL-6 in stimulated saliva (−0.91) was also supported by these results [66]. Wickström et al. [44] also found that the combination of *S. cristatus* and *A. naeslundii,* or independently, did not promote the mucin-degrading activity of dental plaque.

In our microbiome correlation performed in stimulated saliva, *genus Leptotrichia* (0.520), *Capnocytophaga genus* (0.454), and *Fusobacteria* (0.392) play a role in the initial phase of plaque formation, as shown by their positive correlation with melatonin [53].

The *genus Lachnospiraceae* can disappear as caries progresses [55]; however, in the study by Johansson, et al. [67], it was present in Romanian adolescents with a high caries incidence. Its level was correlated positively with MUC5B (0.168) and melatonin (0.021), and based on this, it can be found in patients with good oral hygiene [20]. The *genus Eikenella*, which was found as a part of the core microbiome in the study by Johannsson et al. [67], and *genus Saccharibacteria_(TM7)_[G-5]* were significantly positively correlated with melatonin (*Eikenella:* MUC5B–0.658, melatonin–0.744) and MUC5B (*Saccharibacteria*: MUC5B–0.658, melatonin–0.743). There are contradictory findings about the pathogenicity of *genus Saccharibacteria_(TM7)*; for example, it can be found in a lower ratio in patients with higher caries incidence [68]. The *genus Saccharibacteria_(TM7)_[G-5]* is able to inhibit TNFα [68], which was not supported by our correlation analysis in the case of *genus Saccharibacteria_(TM7)_[G-1]* (0.218) [69].

In line with the study by Wirth et al. [60], the level of *genus Prevotella* was also correlated positively with the levels of IL-1β (0.441), while IL-1β was correlated positively with *Granulicatella* (0.535) and *Rothia* (0.171) [56], in line with our earlier investigation performed in unstimulated saliva. 

The IL-1β and *S. mutans* counts were positively correlated in earlier experiments [15]. Recently, more studies have shown that *S. mutans* is more important in the initiation of the carious process than in its progression [15]. Our present data contradicts these findings, as we found a positive correlation with IL-1β mRNA (0.603) and a negative correlation with IL-1β (−0.463) for *genus Streptococcus*. This can be due to a transcriptional connection between *Streptococci* and IL-1β. The *genus Streptococcus* was found to be the second most frequent in the core microbiome of the stimulated saliva in our study (an average of 0.55), which mainly consisted of non-pathogenic *Streptococci* (*S. parasanguinis clade 411, S. salivarius,* and *S. sanguinis*) based on our previous and present study [27]. These bacteria were found by Wirth et al. [60] mainly in patients with gingivitis [60], which was in line with our investigation, where we found them with a greater log_2_ in the BR group. Contrary to these findings, which indicate that *S. salivarius* has an anti-inflammatory action in the oral cavity [61], it has also been observed in dental caries [58]. Earlier findings showed that MUC7 attaches to *S. gordonii* but MUC5B does not, and *S. sanguinis, S. oralis,* and *S. sobrinus* can bind to MUC7 but not MUC5B [70]. The presence of caries-associated *Streptococci* in the NBR group shows that they can be connected to higher caries prevalence, which is in line with our earlier studies [25].

The biomarkers of a healthy oral cavity [56], *Neisseria* and *Rothia,* were correlated strongly negatively with IL-2 (*genus Neisseria*: −0.579, *genus Rothia*: −0.752).

The IL-6 showed a small positive correlation with *Prevotella* (0.162). In line with the results of Sarkar A [71], *Prevotellas* are able to induce IL-6 secretion. TNFα also showed a positive correlation with *genus Prevotella* (0.607) in stimulated saliva, which is in line with the investigations of Wirth et al. [60], who found *Prevotella* to be pathognomic to gingivitis. 

Baker et al. [56] connected *Prevotellas* with caries and did not find elevated TNFα levels in patients with poor oral hygiene and elevated caries incidence. 

Johansson et al. [67] showed *Prevotellas* in the Swedish caries-free and Romanian caries-active populations, which also strengthens these contradictory findings. Furthermore, in our study population, *Prevotella* was the second most common genus (with an average relative frequency of 0.14) (Figure 12), which was supported by the positive correlation between *Prevotella* and IL-1β, TNFα, and IL-6. These results are in line with the study by Yang, et al. [72], who found that healthy participants and patients with higher caries incidence were characterized by different *Prevotella species*. 

IL-6 was positively correlated with *Haemophilus* (0.518) and negatively with *genus Actinomyces* (−0.415) and *genus Cardiobacterium* (−0.63), which supports the role of IL-6 in good oral hygiene [18].

## 6. Conclusions

Significantly higher levels of MUC5B, MUC7, melatonin, TNFα, IL-1β mRNA, and Ca^2+^ in the stimulated saliva indicate that the production of stimulated saliva through chewing gum without an active ingredient is beneficial in terms of maintaining oral hygiene. 

The positive correlation of MUC7 in the stimulated saliva with bacteria that use mucins as a carbon source (e.g., *Ruminococcae, Bifidobacterium*) or with bacteria that ferment them (e.g., *Bacteriodetes*) confirms the interaction of MUC7 with these bacteria [6].

Correlation analysis between the microbiota, inflammatory mediators, and mucins showed opposite tendencies in the case of the *Haemophilus genus* with mucins and its positive correlation with IL-6. The positive correlation between melatonin and MUC5B was also supported by the microbiota correlation results, as similar trends were observed in the cases of *genus Lachnospiraceae_[G−2]*, *genus Eikenella,* and *genus Saccharibacteria_(TM7)_[G−5]*.

Positive correlation of *Prevotellas* with IL-6, TNFα, and IL-1β supports their role in inflammation as shown in previous studies [56,71]. However, the results of IL-1β [15], contradict its known association with *Streptococcus*, which may be related to the low DMF-T index and other species (e.g., *S. salivarius, S. parasanguinis clade 411*) in our samples. The limitations of the present study include a homogenous population, absence of exclusion for gingival inflammation, and low DMF-T scores.

Nevertheless, the present study adds further evidence to the positive effect of sour cherry on the oral microbiota [25,27,73,74]. Based on the comparison of the cytokine profile, mucin and Ca^2+^ levels, and microbiota composition of the unstimulated and stimulated saliva, it can be concluded that scaling and the subsequent toothbrush exchange have a beneficial effect on the composition of both unstimulated and stimulated saliva. Hence, AC chewing gum has a beneficial effect on the oral health. 

## Figures and Tables

**Table 1:** Primers and probes for the cytokines used in the experiments (Metabion International AG).

Cytokine	Probe (5′-3′)	Forward Primer (5′-3′)	Reverse Primer (5′-3′)
TNFα	CAATGGCGTGGAGCTGAGAG	AACCCCGAGTGACAAGC	TGGGAGTAGATGAGGTACAGG
IL-1β	TGATGGCCCTAAAACAGATGAAGTG	AATTCGGTACATCCTCGA	GATTTTCACCAGGCAAGTCTC
IL-2	TGTGAGCATCCTGGTGAGTTTGGG	AAAGAAAACACAGCTACAACTGG	GAAGATGTTTCAGTTCTGTGGC
IL-6	TGTTACATGTCTCCTTTCTCAGGGC	AATTCGGTACATCCTCGACG	GATTTTCACCAGGCAAGTCTC
GAPDH	CATTGCCCTCAACGACCACTTT	CCTCCACCTTTGACGCTG	CTCTTCCTCTTGTGCTCTTGC

**Table 2:** Characterization of the investigational groups (AG I, AG II) by sex, age, DMF-T (index of decayed, missing, and filled teeth), Dt (number of carious teeth), Mt (number of missing teeth due to caries), and Ft (number of filled teeth).

Group	Participants Number	Sex		Age (M ± SD)	DMF-T (M ± SD)	D (T) (M ± SD)	M (T) (M ± SD)	F (T) (M ± SD)	BPE (M ± SD)
		Female	Male						
AG I	10	7	3	26.8 ± 2.04	4.9 ± 4.38	0.2 ± 0.63	0.8 ± 1.75	5 ± 4.76	0.42 ± 0.33
AG II	10	5	5	36.3 ± 3.83	8.9 ± 4.91	1.1 ± 1.1	1.1 ± 1.37	6.9 ± 3.66	0.33 ± 0.35
ANOVA (*p*)				*p* = 0.0003	*p* > 0.05	*p* > 0.05	*p* > 0.05	*p* > 0.05	*p* > 0.05
Total	20	12	8	31.7 ± 5.71	6.9 ± 4.97	0.65 ± 0.99	0.95 ± 1.54	5.95 ± 4.24	0.42 ± 0.34

**Table 3:** The average of the control values of mucin, cytokine, and melatonin concentrations that can be measured in unstimulated and stimulated saliva (* *p* < 0.0001).

Factor Type of Saliva	MUC5B	MUC7	IL-1β mRNA	IL-1β	TNFα	TNFα mRNA	IL-2	IL-6	Melatonin	Ca^2+^
Unstim.	5.192	0.35	0.208	396.604	22.67	1.101	35.025	10.435	3.76	* 0.431
Stim.	9.477	0.34	0.3	237.34	34.74	1.304	28.698	8.36	7.255	* 1.26

**Table 4:** The most significant differences between the genera in MUC5B and MUC7.

Genus/Mucin	MUC5B	MUC7
*Actinomyces*	0.258	−0.462
*Lachnospiraceae_[G-3]*	0.775	−0.644
*Eikenella*	0.658	−0.301
*Capnocytophaga*	0.250	−0.362
*Porphromonas*	0.415	−0.288
*Peptrostreptococcae_[G-9]*	0.507	−0.431
*Alloprevotellae*	−0.537	0.308
*Bacteriodales_[G-2]*	0.424	−0.19
*Veilonella*	−0.428	0.476
*Bifidobacterium*	−0.284	0.36
*Haemophilus*	−0.641	0.615
*Bacteriodetes_[G-5]*	−0.481	0.517
*Bifidobacterium*	−0.284	0.36
*Mogibacterium*	−0.374	0.242
*Ruminococcae_[G-1]*	−0.1	0.32
*Peptococcus*	−0.311	0.407

## Data Availability

The generated datasets supporting the reported results of the study are available from the corresponding author upon reasonable request.
